# Thrombosis during off pump LVAD placement in a patient with heparin induced thrombocytopenia using bivalirudin

**DOI:** 10.1186/1749-8090-8-115

**Published:** 2013-04-29

**Authors:** Hamdy Awad, Richard Bryant, Obaid Malik, Galina Dimitrova, Chittoor Bhaskar Sai-Sudhakar

**Affiliations:** 1 Department of Anesthesiology, The Ohio State University Wexner Medical Center, Columbus, OH, USA; 2 The Ohio State University College of Medicine, Columbus, OH, USA; 3 The Ohio State University Wexner Medical Center, Columbus, OH, USA

**Keywords:** HeartMate II, Left ventricular assist device, LVAD, Bivalirudin, Stasis, Pharmacology, Off pump, Heparin induced thrombocytopenia, HITT

## Abstract

Here we present our attempt at off pump HeartMate II left ventricular assist device (LVAD) implantation using the anticoagulant bivalirudin in a patient with heparin induced thrombocytopenia, which resulted in thrombosis within the LVAD device. This required that our procedure be converted to on pump, and a new HeartMate II LVAD device to be implanted. In our view, this thrombotic event may have been caused by a number of factors that include bivalirudin’s (1) short half-life of about 20 minutes, (2) decreased activity with blood stasis, (3) inability to prevent clot propagation, and (4) uncertainty with real-time monitoring of therapeutic levels. To prevent future thrombotic events, it may be beneficial to immediately deair the LVAD device prior to the coring of the left ventricle during off pump LVAD placement. In addition, a solution other than blood may be used for priming. If blood is used for priming of the LVAD device, the duration of blood stasis should not exceed 20 minutes when bivalirudin is being used for anticoagulation. Furthermore, this case emphasizes the importance of having a backup LVAD device available and ready to use during surgical procedures.

## Background

Left ventricular assist devices (LVAD) can be implanted with the assistance of cardiopulmonary bypass (on pump), or without cardiopulmonary bypass (off pump). We have recently published our institutional experience with off pump placement of the LVAD [1]. The direct thrombin inhibitor, bivalirudin, has been successfully used for a variety of open heart procedures both on and off pump for patients with heparin induced thrombocytopenia (HIT) [2,3].

We present our attempted case with off pump placement of a HeartMate II LVAD using bivalirudin for anti-coagulation that resulted in clot formation inside the device. This required urgent conversion of the procedure to be completed with cardiopulmonary bypass (CPB) and a new HeartMate II LVAD device was later implanted successfully.

## Case presentation

A 71-year-old Caucasian female with heart failure and a history of heparin induced thrombocytopenia type 2 (HIT) presented for placement of an off pump HeartMate II. Her baseline activated clotting time (ACT) was 124 seconds. A 50 mg bivalirudin bolus was given followed by continuous infusion of bivalirudin titrated to 1.25 mg/kg/hr to achieve an ACT of 326 seconds after induction of general anesthesia and insertion of invasive lines. We have previously described our off pump LVAD implantation technique [4]. A median sternotomy was performed surgically. Aortic and right atrial venous cannulas were placed. A partial occlusion clamp was placed across the ascending aorta and the outflow graft was anastomosed to the ascending aorta and then attached to the device likely to reduce time for initiation of mechanical support after LV apex coring. At this time, the LVAD device was primed and de-aired with retrograde blood flow from the outflow graft for approximately 20 minutes. The ACT at this time was 324 seconds. The heart was fibrillated and the apex was cored out. There was no evidence of the thrombosis in the left ventricular apex, but a fresh clot was noted in the inflow cannula of the LVAD pump, which was flushed out. However, another clot was found inside the LVAD pump that was unable to be flushed out. During this critical time, we urgently converted the procedure to on pump by using CPB. In addition, an additional 40 mg bivalirudin bolus was given, and we increased the infusion rate bivalirudin to 2 mg/kg/hr. The ACT was checked 20 minutes later, and it was 728 seconds. A new LVAD device was subsequently placed. After being weaned from the CPB, the patient became coagulopathic, which was thought to be from the additional dose of bivalirudin and she required significant transfusion of blood products. The patient’s chest was closed with only four wires and she was taken to the Intensive Care Unit after the surgical procedure. She returned to the operating room for mediastinal washout 2 days after her original procedure. She was later discharged from the hospital without complications, and was doing well at home at 16-month follow-up.

## Conclusion

This is our attempted case with off pump LVAD placement reported with bivalirudin, which resulted in significant LVAD thrombosis. In this case, we were able to titrate bivalirudin to increase the ACT to the suggested goal of >300 seconds [5-8]. However, we still had a significant clot burden within the unattached LVAD. Bivalirudin is a direct thrombin inhibitor that has a relatively short half-life of 25 minutes and can be used as an alternative to heparin in patients that have HIT. Bivalirudin has shown procedural success in two open-label, multicenter studies looking at open and off pump CABG [8]. However, no data exists on evidence-based use of bivalirudin in off pump LVAD placement. In our view, this thrombotic event may have been caused by a number of factors that include bivalirudin’s (1) short half-life, (2) decreased activity with blood stasis, (3) inability to prevent clot propagation, and (4) uncertainty with real-time monitoring of therapeutic levels.

The thrombosis may have occurred due to the amount of time that passed (about 20 minutes) between the retrograde priming of the device with blood and the coring of the left ventricle. The timing is important because the half-life of bivalirudin is only 25 minutes [8] (compared to 1.5 hours for heparin). After the device was primed with blood, there was no fresh circulating bivalirudin through the LVAD. This had not been a problem when heparin was used in previous cases for anticoagulation due to its longer half-life. Bivalirudin seems to be the most favorable option amongst the direct thrombin inhibitors with regards to its half-life. However, its short half-life is a double-edged sword. A shorter half-life is beneficial because it allows for quick cessation of effects after discontinuation, but it also leaves the door open for thrombotic complications.

**Figure 1:**
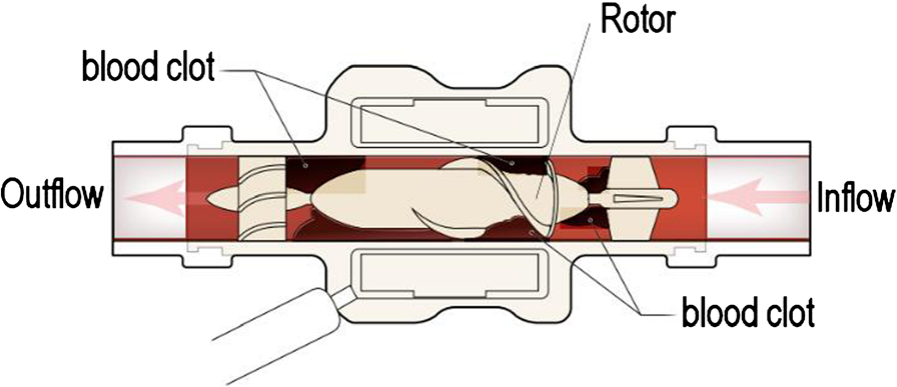
**Stagnant and clotted blood in the HeartMate II device despite adequate anticoagulation with bivalirudin.** The small distance between the rotor and the inner border of the wall may have prevented the clot from being flushed out.

Another plausible mechanism responsible for this clot may be because of bivalirudin’s reversible binding kinetics with thrombin and proteolytic cleavage of bivalirudin by thrombin. Proteolytic destruction by thrombin plays a major role in bivalirudin elimination, and this places areas of blood stasis at high risk of having subtherapeutic bivalirudin levels. Accordingly, several case reports exist of thrombus associated with bivalirudin in CPB venous reservoirs, in the CPB circuit after or near the termination of the CPB procedure, and post cardiotomy ECMO [9,10]. It is possible that there may have been prolonged stasis of blood between the deairing/washout of the LVAD and the coring of the left ventricle, which may have caused a small volume of blood to become stagnant within the HeartMate II device. There is a small distance between the HeartMate II rotor and the inner border of the wall, which may have prevented the clot from being flushed out (Figure 1).

Bivalirudin is unable to prevent clot propagation as effectively as heparin. Nielson et al. have conducted in vitro studies that demonstrate that direct thrombin inhibitors have similar efficacy compared to heparin in initiation of clot formation, but they are not as effective as heparin in preventing clot propagation. Furthermore, ACT utilization to monitor a safe level of anti-coagulation may be grossly inadequate, because ACT only measures clot initiation, and not clot strength or clot propagation [11]. Accordingly, if a clot had formed inside the LVAD, anticoagulation with bivalirudin would have been ineffective in preventing propagation. This may also explain why the clot within the LVAD was not flushed out in this case.

Furthermore, no guidelines exist regarding the optimal dosing or regarding the most prudent method of monitoring the anticoagulation of any of the direct thrombin inhibitors during surgery [4]. Recently, Salemi et al. described a novel approach to monitoring bivalirudin activity by using a specific chromogenic antifactor IIa test. Their study revealed that antifactor IIa assay appropriately shows decreasing bivalirudin plasma levels, while ACT levels remain elevated for an extended period of time. Accordingly, ACT does not have an appropriate sensitivity to detect a drop in plasma bivalirudin concentrations [12]. Even with a continuous infusion of bivalirudin, this could have provided a false sense of security of acceptable anticoagulation in our patient.

It is important for the perioperative team to understand that different implantation techniques can have technical limitations. To prevent this complication in the future and in a patient with HIT, the deairing/priming of the LVAD should be done immediately prior to cannulation of the left ventricle. We also need to be aware of bivalirudin’s pharmacologic properties. A different solution can be used for LVAD priming as opposed to blood. In addition, this case also points out the importance of having a backup LVAD available during surgical procedures.

## Consent

Written informed consent was obtained from the patient for publication of this case report and any accompanying images. A copy of the written consent is available for review by the Editor-in-Chief of this journal.

## Abbreviations

LVAD: Left ventricular assist device; HIT: Heparin induced thrombocytopenia; CPB: Cardiopulmonary bypass; ACT: Activated clotting time.

## Competing interests

The authors declare that they have no competing interests.

## Authors’ contributions

HA conceived of the study, and helped with the drafting and proofreading of the manuscript. RB, OM, and GD participated in background literature review, drafting, and proofreading of the manuscript. CS helped conceive the study, emphasized the significance of this topic, and helped proofread the manuscript. All authors read and approved the final manuscript.
